# Multifunctional co-transport carriers based on cyclodextrin assembly for cancer synergistic therapy

**DOI:** 10.7150/thno.70243

**Published:** 2022-02-28

**Authors:** Shouhui Yi, Rongqiang Liao, Wei Zhao, Yusheng Huang, Yi He

**Affiliations:** 1 Department of Cancer Center, The Second Affiliated Hospital of Chongqing Medical University, Chongqing, 400010, China; 2 Pharmacy Department, Chongqing Emergency Medical Center, Chongqing University Central Hospital, Chongqing, 400014, China

**Keywords:** cyclodextrin, self-assembly, co-delivery, synergistic therapy

## Abstract

In the past few years, drug delivery systems have been used extensively to improve solubility, stability, and pharmacokinetics of chemotherapeutic drugs. However, traditional delivery systems fail to fulfill the required standard of effectiveness, primarily owing to issues of drug resistance exhibited by cancer cells and inherent differences among the patients. In this regard, combination therapy offers advantages of synergistic mechanism, reduced drug dosage, and enhanced therapeutic effect, which can be effectively utilized for the treatment of cancer. However, different types of therapeutic agents exhibit different pharmacokinetic properties and action targets *in vivo*, which lead to uncontrollable concentration ratio of therapeutic agents at the lesion site. This in turn causes serious side effects and affects synergistic anticancer effect. Importantly, multifunctional co-delivery systems are characterized by good pharmacokinetic properties, ability to provide targeted delivery, and controlled release in response to tumor microenvironment. Such delivery systems are widely used for co-delivery of therapeutic agents, which further assist in obtaining better synergistic anticancer effect, and can be potentially used for clinical application. Multifunctional co-delivery systems often exhibit complex structures and construction process. Since cyclodextrin is characterized by self-assembly property, it is possible to quickly construct cyclodextrin-based multifunctional co-delivery systems, with convenience and flexibility. The application of cyclodextrin in the construction of multifunctional co-delivery systems for cancer therapy has gained immense attention in the past few years. The present study provided overview of latest progress in the field of cyclodextrin-based multifunctional co-delivery systems for cancer synergistic therapy.

## 1. Introduction

Cancer is one of the diseases that pose a serious threat to human health 1. The occurrence and development of cancer is regulated by complex signaling network *in vivo* 2. In certain cases, traditionally used treatment methods, such as surgery, radiotherapy, and chemotherapy fail to fulfill the requirements of cancer treatment. Synergetic therapy is a comprehensive treatment strategy that combines two or more medical methods, including chemotherapy, radiotherapy, gene therapy, phototherapy, immunotherapy, and protein therapy. Synergistic therapy has been widely utilized for cancer treatment in the recent years 3. Importantly, synergetic therapy can overcome the issue of drug resistance exhibited by tumors. At the same time, it can improve the safety and effectiveness of the drugs, making it a promising treatment strategy. In addition to this, synergetic therapy can improve patient compliance and reduce the frequency of drug administration 4.

However, differences in physicochemical and pharmacokinetic properties of different types of therapeutic agents might lead to unsatisfactory absorption and distribution 5. In the recent times, nano co-loading has emerged as an ideal strategy, which could effectively avoid these limitations, allowing achievement of maximum therapeutic effect 6. Recent studies showed that nanoparticles played an important role in the improvement of efficiency of co-delivery 7. In particular, nanoparticles can assist in the transportation of drugs, genes, photosensitizers, immunosuppressants, proteins, and other therapeutic molecules. To achieve co-delivery, there is need to develop effective and safe nano carriers 8. In the past few years, nanotechnology provided an efficient platform for the development of intelligent co‐delivery systems, and assisted in the development of various many innovative materials. Multifunctional nano carriers have been widely explored for the delivery and on-controlled release of multiple therapeutic agents at the target site 9.

Self-assembly technology can be particularly used to combine different materials, exhibiting biocompatibility, biodegradability, and negligible cytotoxicity, to construct an excellent nano-delivery platform 10. In particular, self-assembly is similar to the building process of "building blocks", wherein a variety of small molecules are combined to form large molecules. In general, this process does not involve too much outside intervention 11. Interestingly, self-assembly technology, can be used to construct nano delivery platforms, from small to large, from inside to outside, and from bottom to top approaches 12. It has been previously reported that non-covalent interactions play an important role in the process of self-assembly 13. Importantly, elimination of these non-covalent interactions between small molecules would lead to destruction of nano delivery platform. These non-covalent bonds involve reversible bonding form, which includes van der Waals force, hydrogen bond, hydrophobic bond, electrostatic attraction, dipole interaction force, and others 14.

**Figure 1:**
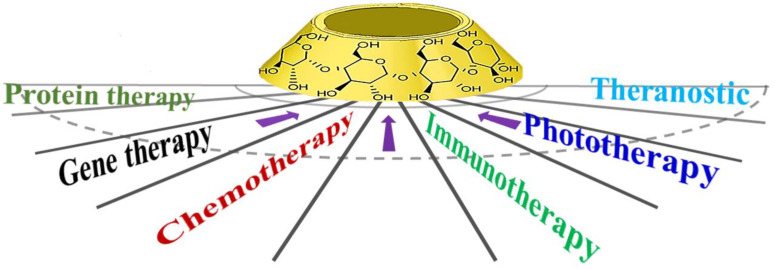
Structure and application of cyclodextrin

Cyclodextrin (CD) refers to a series of cyclic oligosaccharides produced by amylose, under the action of cyclodextrin glucosyltransferase. It usually contains 6-12 *D*-glucopyranose units 15. Among these, molecules with six, seven, and eight glucose units are denoted as alpha-, beta-, and gamma- cyclodextrins, which hold great practical significance 16. Since the glycosidic bond connecting glucose units cannot rotate freely, cyclodextrin exhibits a slightly tapered ring structure instead of cylindrical (Figure 1). In particular, cyclodextrin exhibits a slightly conical hollow cylinder stereoscopic ring structure, wherein outer upper end is composed of secondary hydroxyl groups of C_2_ and C_3_, while lower end is composed of primary hydroxyl groups of C_6_ that are hydrophilic in nature. Importantly, the shielding effect of C-H bond leads to the formation of hydrophobic region in the cavity 17-19. Cyclodextrin is characterized by hydrophobic interior and hydrophilic exterior, which makes it possible to form inclusion complexes and assembly systems with various types of molecules. In particular, these complexes and assembly systems are generated according to van der Waals force, hydrophobic interaction force, and matching between host and guest molecules 20, 21. This selective envelope is generally denoted as molecular recognition, which results in the formation of host-guest inclusion compounds. One of the primary prerequisites for the assembly of cyclodextrin with guest molecule is that the cavity of cyclodextrin must match with the shape of guest molecule. It has been previously shown that cyclodextrin-guest molecule assembly is usually very unstable, primarily owing to kinetics and reversible nature of non-covalent interactions 22. This reversible behavior of cyclodextrin-guest molecule assembly is often utilized to design molecular switches 23. In particular, the assembly process of cyclodextrin and guest molecule offers advantages of being convenient, flexible, and quick. In addition to this, self-assembly offers several other advantages, such as absence of any requirement for special reaction equipment, mild reaction conditions, simple operation, environmental friendliness, and ease of batch preparation 24. It is easy to chemically modify cyclodextrin. In fact, cyclodextrin is known to exhibit low cytotoxicity. Thus, it exhibits broad application prospects in the field of biomaterials, owing to its special characteristics 25. In recent years, other types of macrocyclic compounds, such as cucurbituril, pillararene, and calixarene, have been widely used in drug delivery systems 26, but cyclodextrin exhibits unique advantages compared with these macrocyclic compounds. Cyclodextrin can be obtained naturally and have been widely used clinically as pharmaceutical excipients. Cucurbituril, pillararene and calixarene are artificially synthesized, with harsh synthesis conditions and high synthesis cost. At present, they have not entered clinical application 27. In addition, cucurbituril has poor water solubility and is difficult to be chemically modified 28.

To achieve synergistic therapy, construction of co-delivery systems is particularly imperative. However, most of co-delivery systems involve complex structures, cumbersome construction processes, and low controlled release sensitivity. Therefore, it is necessary to develop a nanocarrier with simple construction process, high delivery accuracy, and effective controlled release. In the recent years, co-delivery systems based on self-assembly of cyclodextrin have been extensively explored, primarily owing to the convenience, flexibility, and rapidity of the assembly process. The present study provided detailed overview of features, mechanisms of action, and potent applications of co-delivery systems based on cyclodextrin. In addition to this, some personal perspectives on this field were also presented.

## 2. Co-transport of different drug combinations

In order to address the issues of non-selectivity and drug resistance associated with cancer, significant efforts have been devoted towards the designing of targeted co-transport nano carriers, which can simultaneously deliver combination of anticancer drugs to tumor lesions and provide synergistic therapy 6, 29. When compared with single drug therapy, combined multi drug therapy does not produce drug resistance easily 30. In recent years, a large amount of co-delivery systems of different drug combinations have been studied (Table 1).

**Figure 2:**
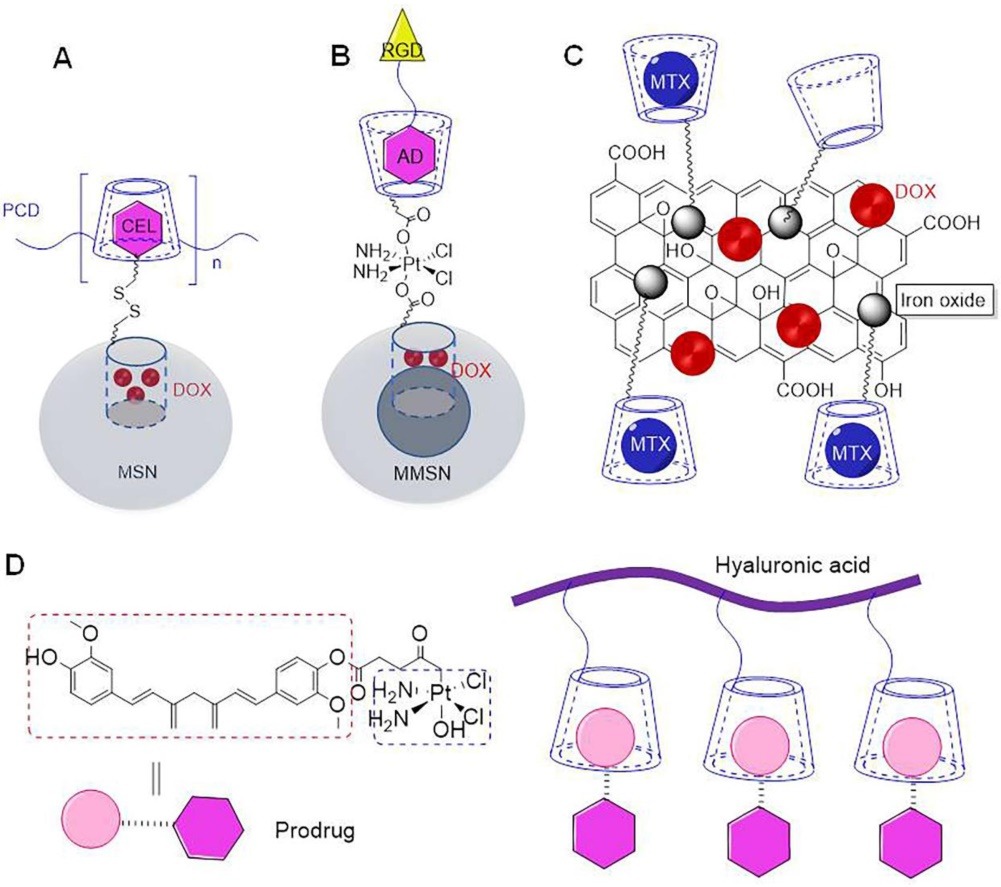
** (A)** Schematic of loading DOX and CEL on MSN 31. **(B)** Schematic of loading DOX and Pt on MMSN 32. **(C)** Schematic of loading DOX and MTX on β-CD grafted magnetic graphene oxide nanocomposites 34. **(D)** Schematic of loading prodrug curcumin-oxoplatin on β-CD modified hyaluronic acid 37.

Cyclodextrin (CD) is often used as a capping agent for mesoporous silica nanoparticles (MSN), primarily owing to its large volume 39. In particular, the hollow tube present inside MSN can be loaded with macromolecular drugs 40. Interestingly, in the absence of any capping agent, drug can easily leak out during transportation. Previous studies reported that poly(β-CD) could adhere to celecoxib-modified MSN, primarily owing to self-assembly of β-CD and celecoxib (CEL) (Figure 2a) 31. In fact, large volume of poly(β-CD), allowed encapsulation of doxorubicin (DOX) in MSN. The study reported that the nanoparticles could effectively co-deliver DOX and CEL to the tumor site. In particular, CEL could inhibit tumor proliferation by blocking COX-2/PGE2 signal transduction, and its combined application with DOX could further enhance anti-tumor effect. It has been previously reported that magnetic mesoporous silica nanoparticles(MMSN) could be used for tumor-targeted magnetic resonance imaging and precise treatment (Figure 2b) 32. The core and shell of these MMSN comprised of Fe_3_O_4_ and MSN, respectively. Importantly, β-CD could be fixed on the surface of MSN shell with the aid of platinum (Pt) to form a capping agent, which could encapsulate DOX in the mesopores. Adamantane modified targeting molecules could be assembled on the surface of the capping agent through host-guest interactions. Interestingly, acidic environment present in the tumor assisted in the separation of capping agent of MMSN, which further assisted in the release of DOX and Pt. In addition to this, it was observed that these nanoparticles showed high contrast in magnetic resonance imaging, under the effect of external magnetic field. In particular, it could magnetically enhance aggregation at the tumor site, which could significantly inhibit tumor growth. Gold mesoporous silica nanoparticles (GMSN) have been previously shown to exhibit excellent co-transport properties, which usually exist in the form of *Janus*. The thiol β-CD could be adsorbed on the surface for gold particles as the carrier of paclitaxel in gold domain, while another MSN with mesoporous structure could be used as the carrier for DOX 33. Importantly, *Janus* GMSN exhibited dual response release characteristics towards pH and near infrared. Thus, the study demonstrated that dual drug-loaded *Janus* GMSN exhibited high therapeutic efficiency and excellent biocompatibility, both *in vitro* and *in vivo*. Consequently, these nanoprticles be used as effective candidates for cancer co-therapy.

β-CD grafted magnetic graphene oxide nanocomposites are known to have composite properties of cyclodextrin, iron oxide, and graphene (Figure 2c) 34. The β-CD in the nano carrier could simultaneously load DOX and methotrexate (MTX), to assist in synergistic treatment of tumors. These nanoparticles could also add other components. Manita Das et al. reported the construction of new nanoparticles using β-CD, hydrogels, fluorescent compounds, and iron oxides 35. Hydrophobic drugs could be assembled into β-CDs, while hydrophilic drugs were assembled in hydrogels. Importantly, fluorescent groups enabled monitoring of the process of cellular phagocytosis of nanoparticles, while iron oxide could achieve targeted effect.

In addition to simultaneous assembly and transportation of two independent drugs, a prodrug can be produced by connecting two drugs that can further assembled with CD to ensure simultaneous transport. Aminosalicylic acid is known to exhibit anti-inflammatory properties. It has been previously shown to reduce the risk of colorectal cancer 41. Histone deacetylase has been shown to accelerate the progression of colon cancer. Butyric acid is a histone deacetylase inhibitor, which has been previously shown to exert an inhibitory effect on the progression of colon cancer. However, butyric acid is associated with limitations of volatility and corrosiveness 42. It has been previously shown that these shortcomings could be effectively overcome by conjugating butyric acid with aminosalicylic acid to form prodrug, which was further assembled with folic acid modified β-CD 36. When compared with a single drug, the assembly formed by folic acid modified β-CD and prodrug exhibited a better inhibitory effect on tumors *in vivo* and *in vitro*. Bai et al. conjugated curcumin with oxoplatin to form a prodrug, and then assembled it with β-CD modified hyaluronic acid to construct nano drug carrier (Figure 2d) 37. In particular, prodrugs could be assembled with β-CD mainly via non-covalent interactions between curcumin and β-CD. The inclusion ratio of curcumin and cyclodextrin is 1:1. Science curcumin and oxoplatin were connected by ester bond, acidic environment triggered breakage of internal ester bond, which would lead to release of curcumin and oxoplatin. The cross-linked β-CD and γ-CD polymers exhibited multiple cavities of different sizes, such that two drugs of different sizes could be transported. Bognanni et al. reported preparation of cross-linked β-CD and γ-CD polymers through epichlorohydrin, wherein targeted modification of the surface of CD enabled targeting function 38. This cross-linked polymer could assemble DOX and oxaliplatin at the same time.

Currently, designing of advanced controlled release system is a key topic in the field of drug carrier. Combination medication might exert a significant impact on the efficacy and toxicity of chemotherapy, primarily owing to drug interactions or cycle specificity of anti-cancer drugs. Therefore, a correct drug releases sequence would not only increase anti-tumor efficacy, but it would also reduce toxic side effects 43. For example, when paclitaxel was used in combination with cisplatin, cisplatin delayed the excretion of paclitaxel, and aggravated adverse reactions 44. Thus, paclitaxel must be given first followed by cisplatin. In particular, the slower-growing tumors are characterized by a longer G_0_ stage, and cycle non-specific drugs must be applied first. Following this, cycle-specific drugs should be applied, after the tumor is induced to enter proliferation phase. Completely opposite trend is followed in case of faster-growing tumors. Villalonga et al. designed a novel nanomachine for sequential release of two different compounds 45. The nanoparticles were grafted with sulfhydryl groups and pH-sensitive β-CD gated integration on the surface of gold mesoporous silica modified by acetylcholinesterase. These nanoparticles exhibited the mechanism of enzyme controlled and stimulated reaction release, and could selectively release different drugs following addition of enzyme substrate. The nanoparticles also allowed a time-controlled sequence for the release of encapsulated drug.

## 3. Co-transport of drug and regulatory gene

Combined application of multiple mono-therapies is known to enhance therapeutic efficacy of each other. Such a therapy results in a significant super additive effect, wherein "1 + 1 > 2" 46. Drug resistance is one of the key factors that are associated with failure of clinical treatment and recurrence. The main contributing factor involves abnormal expression of proteins, such as efflux pumps, glycoproteins, DNA repair enzymes, and replicase, which are encoded by drug resistance genes. Combination of drug and regulatory gene has been widely used for intensive treatment of cancer, primarily owing to its potential synergistic effect 47. The co-delivery of drug and regulatory gene using nanocarriers can be considered as an important strategy to enhance the efficiency of anticancer therapy. In recent years, a large amount of co-delivery systems of drug and regulatory gene have been studied (Table 2).

**Figure 3:**
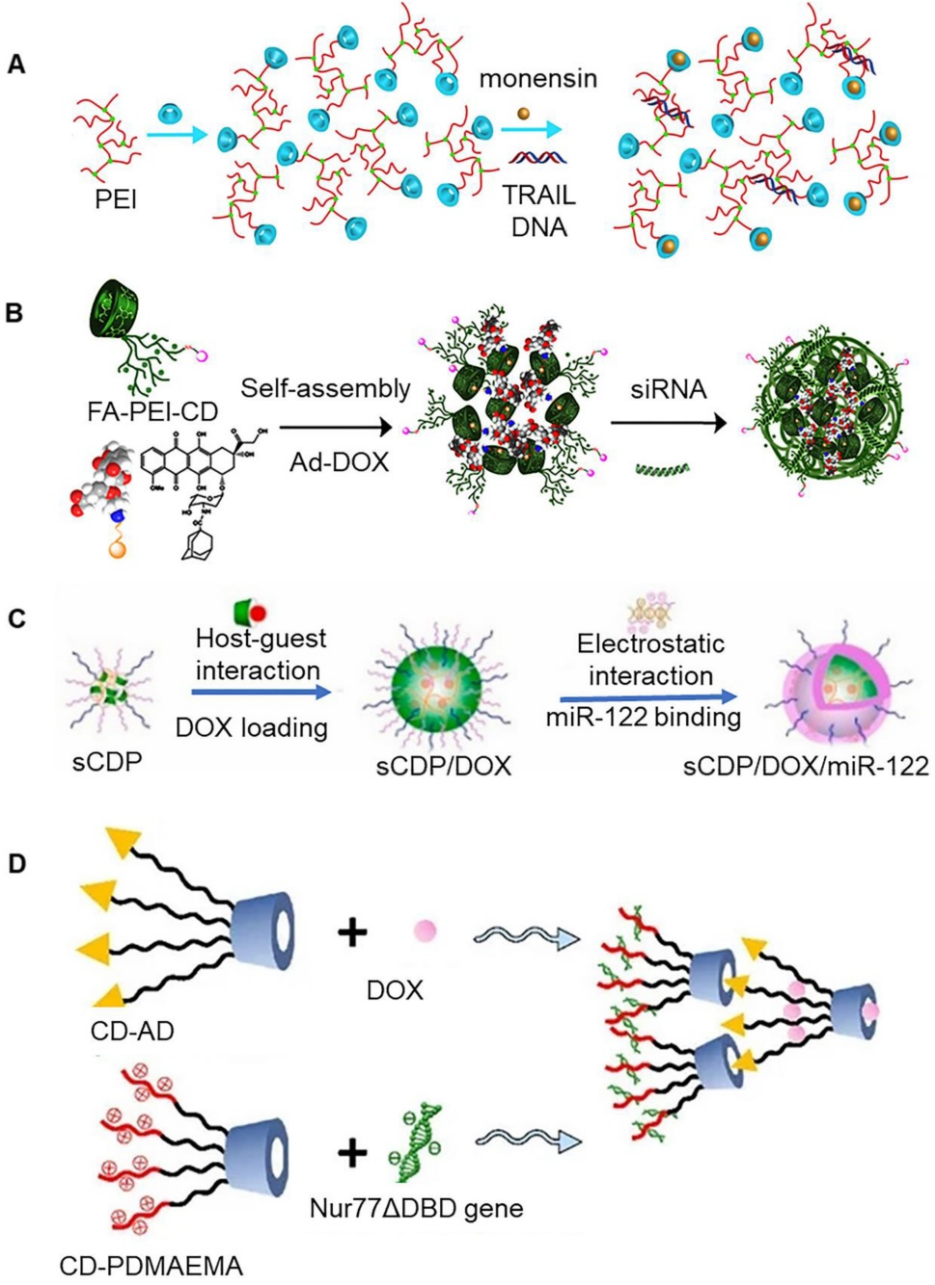
** (A)** Co-delivery system of *TRAIL DNA* and monensin. Adapted with permission from 48, Copyright 2018 Elsevier. **(B)** Co-delivery system of DOX and *siRNA*. Adapted with permission from 49, Copyright 2022 Elsevier. **(C)** Schematic of loading DOX and *miR-122* on cyclodextrin-based star copolymer nanoparticle. Adapted with permission from 50, Copyright 2021 Elsevier. **(D)** Schematic of loading DOX and *Nur77ΔDBD* gene on double-layer nanoparticles. Adapted with permission from 53, Copyright 2021 MDPI.

Tumor necrosis factor related apoptosis-inducing ligand (*TRAIL*) is a member of tumor necrosis factor superfamily, which is known to selectively induce tumor cell apoptosis. It has been previously shown to be non-toxic to normal cells 55. It is expected that this molecule will emerge as a new method for tumor treatment. In fact, it has attracted widespread attention. The co-delivery system of *TRAIL DNA* and death receptor sensitizer (monensin) could be assembled by β-CD (Figure 3a) 48. Polyethyleneimine (PEI) could connect multiple β-CDs simultaneously through disulfide bonds, wherein β-CD hydrophobic cavity could be used to load monensin, while PEI could tightly bind to *TRAIL DNA*. These disulfide crosslinked nanoparticles were redox-sensitive, and their rapid disassembly in tumor cells facilitated release of the regulatory gene and drug. Further studies showed that this co-transport system could overcome tumor resistance. In addition to this, PEI could also be attached to only one β-CD, which could be further aggregated with each other to co-delivery drug and *siRNA* (Figure 3b) 49. The adamantane-modified doxorubicin (Ad-DOX) conjugate could be assembled with PEI-CD to form nanoparticles using host-guest interaction. The surface of PEI-CD could be further modified with folic acid (FA) targeting molecules to provide tumor targeting ability.

In addition to PEI, which is often used as a regulator gene carrier, other cationic polymers can also be used as carriers. Xiong et al. reported assembly of a β-CD-cored star copolymer nanoparticle (Figure 3c) 50. β-CD-cored star polymer was characterized by presence of poly (2-(dimethylamino) ethyl methacrylate) (PDMAEMA) and hydrophilic poly(ethylene glycol) (PEG) arms. In this nano-system, *miR-122* and DOX were encapsulated inside, providing a sequential release of* miR-122* and DOX. The preferential release of miR-122 directly induced cell apoptosis by down regulation of Bcl-w and enhanced p53 activity. At the same time, it also increased DOX accumulation via inhibition of cytotoxic efflux transporter expression, which ensured synergistic performance on cell inhibition. These nanoparticles displayed a remarkable increase in anti-tumor efficacy *in vivo* as compared to free DOX, highlighting its potential to be utilized in hepatoma therapy. A previous study reported that hyperbranched poly(amido amine)s (PAAs) could be used as a skeleton material to introduce β-CD. These nanoparticles exhibited similar properties to carry tissue factor pathway inhibitor 2 (*TFPI-2*) and docetaxel (DOC) at the same time 51. The conventional modification of β-CD mainly occurs on its primary face. However, it's the secondary face can also be modified. Zou et al. conjugated primary and secondary faces of β-CD to hydrocarbon chains (C_12_) and polar (propylamino) groups, respectively, to form amphiphilic compounds. Further, these amphiphilic compounds aggregated with each other to produce docetaxel (DTX) and *siRNA* carriers 52.

β-CD-adamantane (β-CD-Ad) and β-CD-poly(2-(dimethylamino)ethyl methacrylate) (β-CD-PDMAEMA) could be assembled into double-layer nanoparticles (Figure 3d) 53. The inner layer and outer layer were assembled together via interactions between β-CD and adamantane. The inner layer of the nanoparticles was involved in the assembly DOX, and PDMAEMA present on the surface of outer layer could bind *Nur77ΔDBD* gene. The protein expressed by *Nur77ΔDBD* gene is known to be involved in the inhibition efflux pump, which can prevent elimination of drug from the tumor cells by the efflux pump. Integrating of β-CD-modified polyamidoamine dendrimers with MSN could result in the generation of a multifunctional carrier for co-transport of drugs and *siRNA* 54. Here, the dendrimers and MSN were connected by disulfide bonds. In particular, MSN was involved in loading of macromolecular chemotherapeutic drugs (SN-38), dendrimers were involved in binding to *siRNA*, and β-CD on the surface of dendrimers could assemble small molecular chemotherapeutic drugs. In addition to this, β-CD could also be tightly combined with the 4T1 cancer cell membrane to stabilize the multifunctional co-transport system.

Although co-delivery carriers have made major breakthroughs in the past few years, some technical problems still need to be resolved. In particular, there is need to further optimize design strategy for co-delivery carriers to achieve best combined anti-cancer effect. Most of the co-delivery carriers are known to simultaneously release drugs and genes in the same organelle of cancer cells. In fact, carriers cannot provide independent release methods for drugs and genes. Such release behavior fails to coordinate the mechanism of action of drugs and genes, which further limits the synergistic effect of the two. Therefore, it is necessary to design and develop a co-delivery system that can provide sequential release of drugs and genes at different times and locations. In addition to this, a series of problems need to be solved. In particular, one must identify suitable strategies to choose right combination of drugs and genes, to determine optimal use ratio of drugs and genes, to improve biocompatibility and serum stability of the carrier, and to avoid adverse immune stimulation caused by combined administration.

## 4. Co-transport of drug and photosensitizer

Phototherapy mainly includes photodynamic therapy (PDT) and photothermal therapy (PTT) 56. The basic principle for photosensitizer uptake by pathological tissue involves production of cytotoxic reactive oxygen species (ROS) or heat under light, causing irreversible cell damage and induction of cell death 57. Phototherapy occupies an important position in the field of nanomedicine primarily owing to the advantages of non-invasiveness, good targeting ability, absence of drug resistance, short treatment time, and synergy with chemotherapy 58. In the past few years, strategic combination of phototherapy and chemotherapy has emerged as a promising treatment option for various tumor indications, which expanded current understanding regarding each individual modality, and identified new opportunities to achieve super-additive benefits via exploration of their internal synergy rather than simple mixing 59. In the recent years, a large amount of co-delivery systems of drug and photosensitizer have been studied (table 3).

### 4.1 Photodynamic therapy and chemotherapy

**Figure 4:**
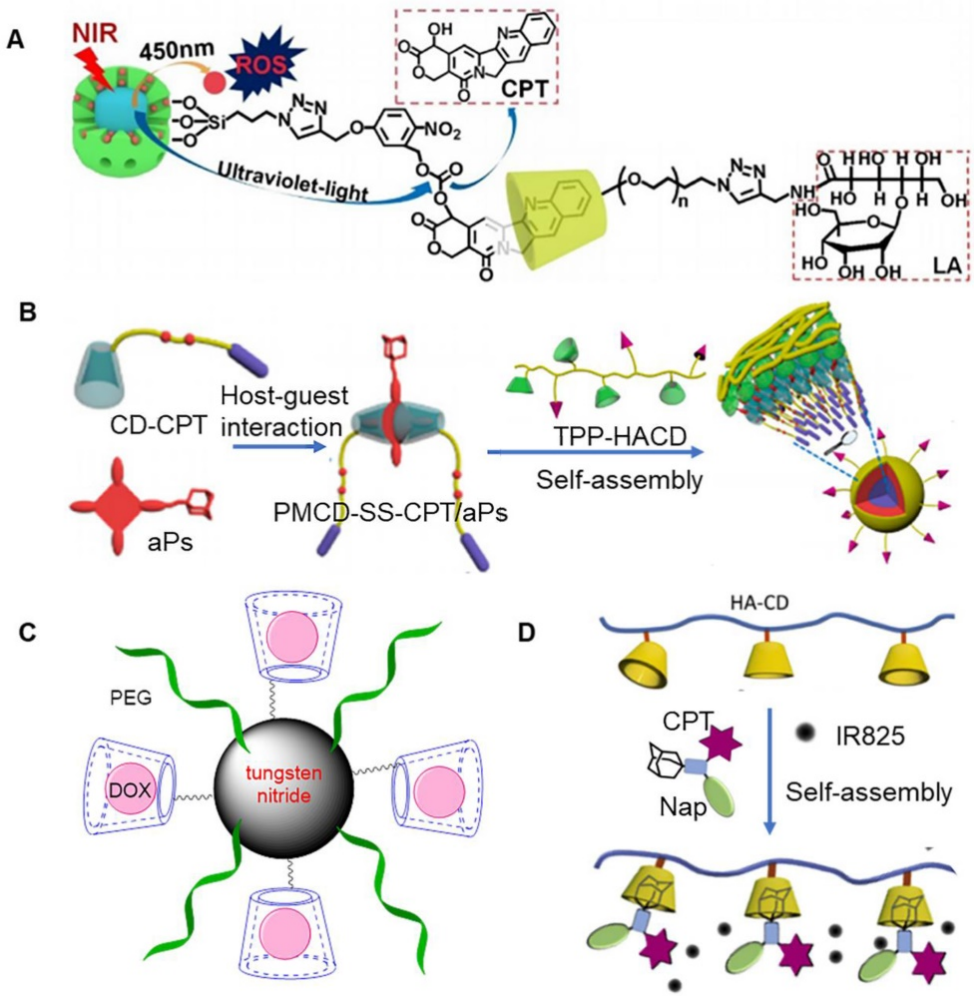
** (A)** Schematic of dual-emissive upconverting nanoparticle. Adapted with permission from 60, Copyright 2020 Elsevier. **(B)** Assembly process of prodrug (CD-CPT) and adamantane-porphyrin photosensitizer (aPs). Adapted with permission from 62, Copyright 2020 American Chemical Society.** (C)** Schematic of tungsten nitride modified by PEG and β-CD 63. **(D)** Assembly process of β-CD-functionalized hyaluronic acid and Nap-CPT-Ad. Adapted with permission from 64, Copyright 2018 Elsevier.

It is well established that most of the currently existing photosensitizers must be excited using ultraviolet light to initiate a photochemical reaction, which might increase the risk of ultraviolet-related side effects and would affect the availability of PDT for clinical application 66, 67. A promising strategy to overcome the side effects associated with photosensitizers involves the use upconverting nanoparticles (UCNPs) as intermediate energy donors. UCNPs are usually composed of inorganic molecules that are doped with lanthanides. It has been previously shown that these nanoparticles could emit ultraviolet-visible light through anti-Stokes under near-infrared excitation 68, 69. Near infrared light is known to exhibit less phototoxic side effects as compared to ultraviolet and visible light, and offers advantage of deep tissue penetration 70, 71. Luo et al. reported the construction of a new type of dual-emissive upconverting nanoparticle (UCNP) with *β-NaYF_4_·0.5%Tm^3+^·30%Yb^3+^/β-NaYF_4_* core and MSN shell (Figure 4a) 60. In particular, MSN could be loaded with photosensitizer (1,8-dihydroxy-3-methylanthraquinone, DHMA), and the surface could be conjugated with UV-activatable camptothecin (CPT) prodrug. Further, lactobionic acid modified-β-CD could be combined with CPT prodrug through host-guest interactions. It was observed that lactobionic acid modified-β-CD enhanced the aqueous dispersity of the nanocarriers and prevented DHMA leakage. At the same time, it also improved the targeting effect on tumor cells. These nanoparticles could emit UV (360 nm) and visible light (480 nm), when excited using infrared light (980 nm). In particular, UV could break chemical bonds present within the prodrug, resulting in release of cytotoxic CPT, and visible light could excite the photosensitizer to produce ROS.

Zinc phthalocyanine is another common photosensitizer. Its clinical application is limited owing to its hydrophobicity. Zinc phthalocyanine modified by polyethylene glycol (ZnPc-(PEG)_5_) can be used as an amphiphilic material for tumor therapy. A previous study reported that β-CD modified acetonization and DOX could form an assembly. Following this, ZnPc-(PEG)_5_ was adsorbed onto the surface of the assembly to form nanoparticles, which could provide synergistic treatment for tumors 61. In particular, ZnPc-(PEG)_5_ could adhered to the surface of β-CD modified acetonization, due to hydrophobic interactions. An acidic environment triggered the decomposition of the nanoparticles, which eventually lead to the release of the drug. Under near-infrared, the nanoparticles exhibited relatively high efficiency to generate ROS. Cell viability and apoptosis assays showed that chemotherapy and PDT exhibited synergistic cytotoxic effects. Another study demonstrated that β-CD could be connected with camptothecin (CPT) to form prodrugs, which further assembled with photosensitizers to form nanoparticles, that could be used for synergistic therapy (Figure 4b) 62. Prodrug (CD-CPT) could be assembled simultaneously on both sides of adamantane-porphyrin photosensitizer (aPs) to form spindle like macromolecules. This process was realized by the interaction between host and guest, in which host and guest were present at the ratio of 2:1. Free adamantane present on the surface could also be assembled with hyaluronic acid-β-CD to produce micelles with tumor targeting effects. Under the effect of light of a specific wavelength, porphyrin absorbed energy and reacted with ROS, which directly caused cell damage and even cell death. The production of ROS further destroyed the chemical bonds present between β-CD and drug, which eventually lead to drug release. Introduction of photosensitizer usually involves a clever designing strategy, which should promote the release of drugs, and kill tumor cells.

### 4.2 Photothermal therapy and chemotherapy

Under near-infrared light irradiation, some photosensitizers have been shown to rapidly generate heat, which could effectively induce tumor thermal ablation 72. Recent studies reported extensive use of tungsten nitride, a kind of black metal photocatalyst, in tumor hyperthermia, primarily owing to its biocompatibility and thermogenesis under infrared light irradiation. It has been previously reported that tungsten nitride modified by PEG and β-CD possessed strong near-infrared absorbance, high photothermal conversion efficiency, and excellent photothermal stability. Additionally, it could effectively inhibit the growth of tumor cells, when irradiated with 808 nm infrared light (Figure 4c) 63. β-CD could be loaded with DOX, which could produce synergistic effect with tungsten nitride. when the assembled system was exposed to light for 8 minutes (1 W/cm^2^), the temperature of the solution increased to ~25°C. The assembly system exhibited an inhibition rate of ~85% on CT26 cells under light irradiation, which was significantly higher as compared to non-light conditions. In addition to this, the assembled system presented good contrasting capability for X-ray computed tomography (CT) and photoacoustic (PA) imaging. Following introduction of tumor targeting molecules on the surface of nanoparticles, the tumor could be accurately positioned for precise treatment through CT/PA imaging.

In case of traditional drug delivery systems, *in vitro* release process for drugs could be hardly visualized, which is important accurate and effective drug delivery. In a previous study, it was observed that once naphthalimide and CPT were connected to adamantane with nitrobenzene through disulfide bonds (Nap-CPT-Ad), the fluorescence of these two chromophores could be quenched due to light-induced electron transfer mechanism (Figure 4d) 64. It was reported that β-CD-functionalized hyaluronic acid and Nap-CPT-Ad could be self-assembled to form nanoparticles through host-guest interactions, which could also be loaded with near-infrared absorbing dye IR825. Importantly, disulfide bond present in Nap-CPT-Ad were cleaved in a reducing environment, resulting in the release of coupled drug and recovery of fluorescence emission. The release process of the drug was visualized under a fluorescence microscope. At the same time, the dye IR825 effectively converted the absorbed light into local heat, making nanoparticles an effective photothermal treatment system. Since it is easy to chemically modify CD, cisplatin can simultaneously bind two CDs to form prodrugs (Pt-CD). A previous study reported that adamantane-modified lipid pyridine amphiphiles could be assembled with Pt-CD to form liposome nanoparticles, through host-guest interactions between β‐CD and adamantane 65. When loaded with IR780, the nanoparticles possessed chemotherapeutic and photothermal effects. Under near‐infrared light, the photothermal effect of nanoparticles could induce mitochondrial damage in cancer cells and strengthened chemotherapeutic efficacy of cisplatin, such that it could finally realize the combined anticancer treatment based on mitochondrial damage.

As a "focus on point" treatment, phototherapy is known to produce cytotoxic substances locally, only under the effect of external excitation, and thus could avoid systemic toxicity caused by off-target problems. Consequently, it has become a promising treatment method. Although phototherapy has developed rapidly in the field of antitumor research, most of these methods still cannot be applied to clinical transformation. This is primarily attributed to defects of photosensitive materials in tumor treatment 73. In particular, photodynamic therapy produces reactive oxygen species with short lifetimes and effective diffusion distances, while photothermal therapy can cause thermal damage to normal tissues. In addition to this, the penetration effect of excitation light source is usually poor, and photosensitizer is less sensitive to light in case of deep tissues. These deficiencies further limit the widespread application of phototherapy.

## 5. Co-transport of regulatory gene and photosensitizer

In the past few years, gene therapy has emerged as a promising treatment method for cancer 74. To achieve high efficiency gene therapy, it is important to design safe and effective gene delivery carriers. Gene therapy is mainly limited owing to contradiction between transfection efficiency and material toxicity, which can be resolved by development of stimulus‐responsive carriers that can controllably degrade materials and release therapeutic genes 75. Light serves as a highly adjustable exogenous stimulus. Importantly, irradiation position, intensity, duration, and wavelength of the light can be accurately controlled in real time by light source. A photosensitizer can absorb light and convert it into ROS, which can be further used as a trigger for gene release. In addition to this, the generated ROS can directly kill cancer cells. However, the co-delivery systems based on cyclodextrin for regulatory gene and photosensitizer has not been widely studied (Table 4).

**Figure 5:**
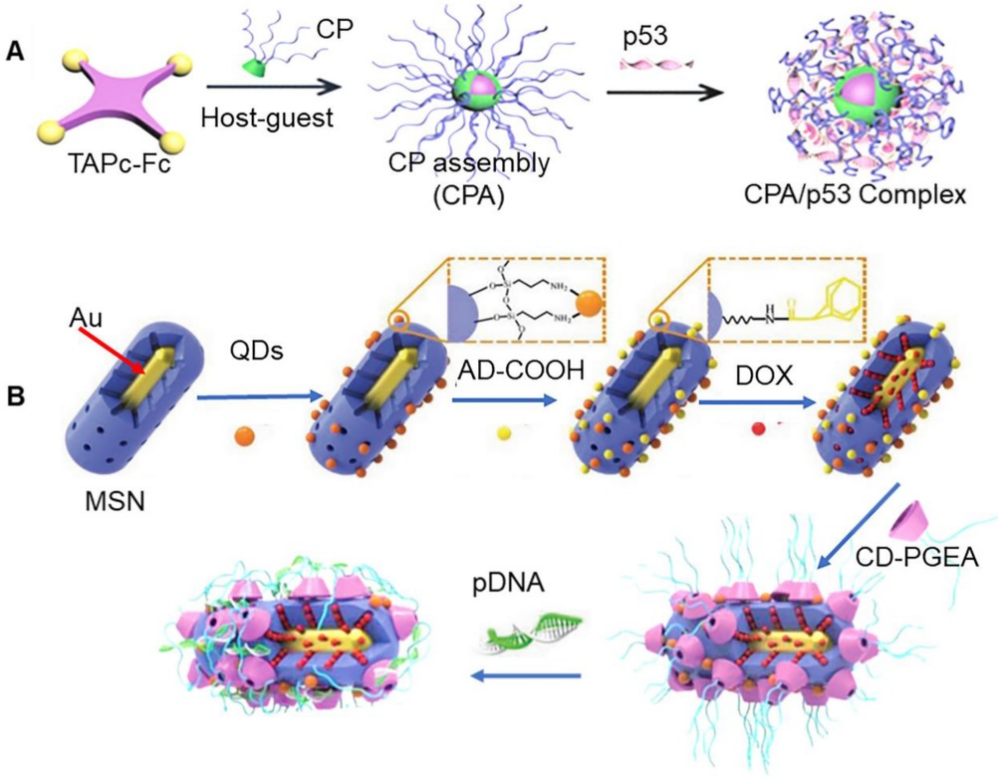
** (A)** Assembly process of β-CD based polycations and Fc-functionalized zinc tetraaminophthalocyanine. Adapted with permission from 76, Copyright 2019 Wiley-VCH. (B) Schematic of multifunctional nanoparticle with imaging, drug therapy, gene therapy and hyperthermia functions. Adapted with permission from 79, Copyright 2017 Wiley-VCH.

Ferrocene (Fc) and β-CD are known to exhibit good space matching and hydrophobic interaction, which allows easy formation of assembly. In particular, the assembly can be decomposed upon exposure to oxidative conditions, which makes it an outstanding candidate for cancer treatment. A previous study reported that nanoparticles assembled by β-CD based polycations and Fc-functionalized zinc tetraaminophthalocyanine (TAPc-Fc) could be used for regulatory gene and photosensitivity synergistic therapy (Figure 5a) 76. Reactive oxygen could accelerate the decomposition of the assembly complex, which further promoted the release of *p53* gene. Fc could be oxidized to water-soluble Fc^+^ after exposure to H_2_O_2_, which could accelerate the detachment of the Fc^+^ unit from the hydrophobic inner cavity of β-CD, resulting in eventual disintegration of the assembly. It has also been previously shown that β-CD and tetraphenylporphyrin (TPP) could self-assemble through benzene ring of TPP and hydrophobic cavity of β-CD. Chen et al. reported construction of novel nanoparticles via non-covalent interactions between PEGylated TPP and β-CD-modified poly(L-lysine) 77. Interestingly, TPP could produce singlet oxygen under light irradiation, which further resulted in increase in ROS and inhibition of tumor cells. Killerred protein is a new photosensitizer, which is known to produce ROS under green light irradiation. It has been shown to exhibit broad application prospect in tumor therapy. A previous study reported that transfection of plasmid Killerred (*pKR*) allowed tumor cells to express Killerred protein on their own for photodynamic therapy, which avoided immune response associated with direct protein delivery. Xu et al. used β-CD modified poly (glycidyl methacrylate)s and adamantane functionalized hydroxyethyl starch backbone to build carriers for plasmid *pKR* and *p53* transportation. In particular, *pKR-p53* could express killerred and *p53* proteins, which were used in photodynamic therapy, and *p53* mediated apoptosis therapy, respectively 78.

Gold nanomaterials are known to exhibit unique optical properties. In fact, these nanoparticles can also be directly excited to generate singlet oxygen. Since gold nanoparticles exhibit high resistance towards oxidative decomposition by ROS oxidation, these nanoparticles can be directly used as photosensitizers 81. In addition to this, gold nanoparticles can efficiently absorb near-infrared light and convert photons into thermal energy, such that they can be used for thermal ablation of tumors. In a previous study, Xu et al. produced a multifunctional nanoparticle, with imaging, drug therapy, gene therapy, and hyperthermia functions using gold nanorods, mesoporous silica, quantum dots, and β-CD modified poly (glycidyl methacrylate) (CD-PGEA) (Figure 5b) 79. MSN integrated Au with quantum dots to build nano-frames. The X-ray opacity properties of Au could be used for tomography (CT) imaging, while fluorescent quantum dots further enhanced imaging sensitivity. The hollow mesopores of MSN were loaded with DOX and sealed using *pDNA*/polycationic β-CD complex. Importantly, Au could be used in photothermal therapy. This photothermal effect also promoted separation of capping agents, which triggered the release of drugs and genes. In addition to this, polycationic β-CD could also be directly adsorbed onto the surface of Au nanorods, and then assembled with therapeutic genes to form nanocarriers for photothermal therapy and gene therapy 80.

Phototherapy offers advantages of controllability and non-invasiveness. At the same time, phototherapy can also trigger stress protection mechanism in cancer cells, which involves increase in heat shock proteins, expression of various pro-survival factors, and increase in angiogenesis signaling molecules, which further lead to increased tolerance of cancer cells towards phototherapy. This in turn limits the efficacy of phototherapy 82, 83. Besides this, phototherapy is known to generate heat, which affects the stability of genes 84. In case gene gets deteriorated under the effect of light, co-transport system would loses the value for co-treatment 85. Therefore, newly constructed carriers should enhance the sensitivity of photosensitizers and increase the stability of genes.

## 6. Co-transport of immunopotentiator and others

Nanomedicine has been shown to exhibit great potential to improve the efficacy of cancer immunotherapy 86. Nanomedicine usually aims to improve direct killing of cancer cells, by improving the delivery of chemotherapeutic drugs to tumors, and metastases. Various nano drug preparations have been explored to enhance anti-cancer immunity and cooperate with clinically established immunotherapy 87. In the recent years, a large amount of co-delivery systems of immunopotentiator and drug (protein or photosensitizer) have been studied (Table 5).

**Figure 6:**
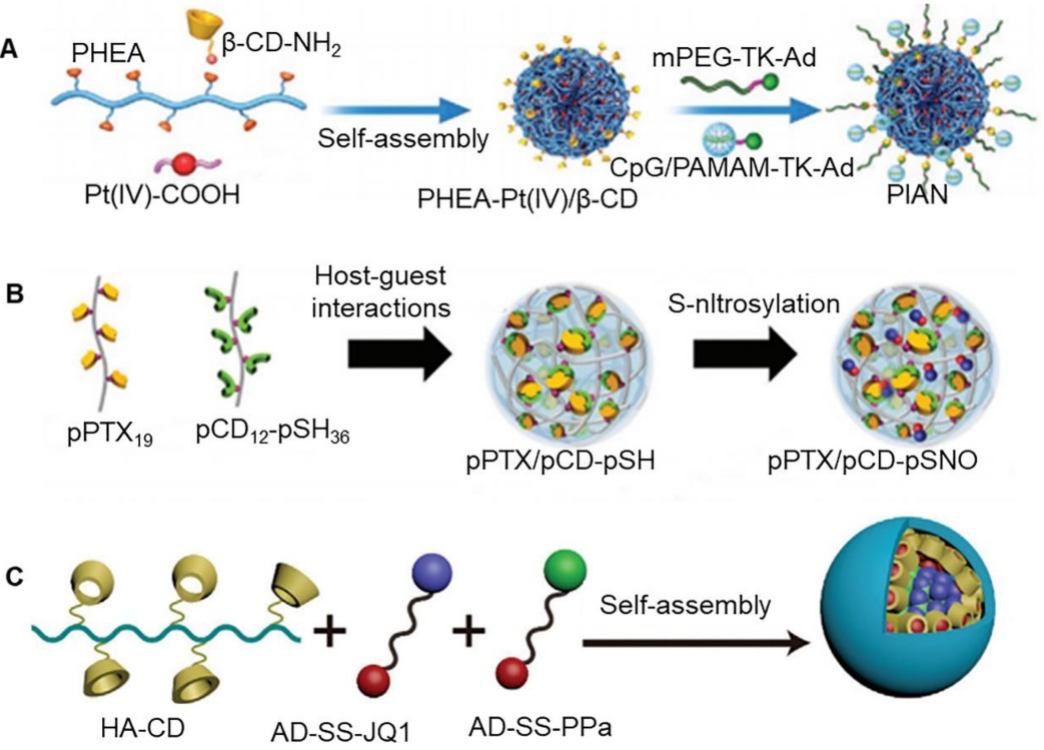
** (A)** Assembly process of three kinds of blocks: PHEA-Pt(IV)/β-CD, CpG/PAMAM-TK-Ad and mPEG-TK-Ad. Adapted with permission from 88, Copyright 2021 Wiley-VCH. **(B)** Schematic of nanoparticle based on the assembly of polymeric paclitaxel and polymeric cyclodextrin containing nitric oxide. Adapted with permission from 92, Copyright 2020 Wiley-VCH. **(C)** Schematic of supramolecular prodrug nano-platform. Adapted with permission from 93, Copyright 2021 Wiley-VCH.

Immunotherapy, especially immune checkpoint inhibitor therapy, has completely revolutionized the clinical treatment of cancer 94. However, it is quite difficult to use simple nanoparticles to fulfill all the requirements of complex immune activation process. Thus, multifunctional nanoparticles can be constructed by host-guest interaction of cyclodextrin, which can simultaneously carry chemotherapeutic drugs and immunopotentiators into tumor cells. A previous study reported construction of nanoparticles from assembly of three kinds of blocks, namely poly-[(N-2-hydroxyethyl)-aspartamide]-Pt(IV)/β-CD (PHEA-Pt(IV)/β-CD), CpG/polyamidoamine-thioketal-adamantane (CpG/PAMAM-TK-Ad), and methoxy poly(ethylene glycol)-thioketal-adamantane (mPEG-TK-Ad) (Figure 6a) 88. CpG is a powerful adjuvant that induces maturation of dendritic cells. It could be loaded into nanoparticles through self-assembly of β-CD and adamantane. High levels of ROS in the tumor microenvironment decomposed the nanoparticles, which promoted release of Pt and CpG. Pt could kill tumor cells and caused antigen release. Further, CpG carried out a series of immune processes, which included loading of antigen from apoptotic tumor cells through electrostatic adsorption, carrying antigen into lymphocytes, and triggering internalization of antigen by dendritic cells. The exposed CpG in the cells promoted maturation of dendritic cells and presented antigen to T cells, thereby generating a strong anti-tumor immune response 95. Interestingly, host-guest interactions of β-CD could be used to assemble, chemotherapeutic drugs, photosensitizers, and immunomodulators to form a multifunctional nanomedicine, which could effectively control tumor progression, metastasis, and recurrence 89. In a previous study, Immunomodulator CpG, polyethylene glycol (PEG) chains, and dendrimer polyamide were linked by chemical bonds to form macromolecular polymers (CpG-P-ss-M), which were added to CD, DOX, and indocyanine green (ICG) to obtain multifunctional nanoparticles. Under light irradiation, ICG generated heat and induced tumor cell apoptosis. In addition to this, heat damaged the gel system, resulting in the release of DOX and CpG.

Tyrosinase-related protein 2 (Trp2) has been widely studied as a classic melanoma antigen. In particular, it has been shown to enhance the activity of antigen-specific cytotoxic T lymphocytes *in vivo* 96. As an agonist, imiquimod (R837) can significantly promote the activation of T cells and inhibit the occurrence of tumors. However, its hydrophobicity limits its wide application. A previous study reported that mannosylated-cyclodextrin could pack R837 into hydrophobic cavity (Man-CD/R837). Negatively charged sodium alginate is a biocompatible polymer. It could combine Man-CD/R837 and Trp2 to form nanoparticles through physical adsorption 90. It was observed that the resulting nanocomposite could be taken up by the cells, and it showed the effect of enhanced the secretion of Th1 cytokines. In another study, folic acid targeting polyethylene glycol modified amphiphilic β-CD nanoparticles were shown to assemble ginsenoside Rg3 and quercetin 91. This co-delivery system could improve drug delivery barrier in the body. In particular, ginsenoside Rg3 could induce immunogenic cell death of colorectal cancer cells, while quercetin triggered the production of ROS, which enhanced the efficacy of ginsenoside Rg3 in inducing immunogenic cell death. Thomas et al. reported the development of a nanoparticle based on the assembly of polymeric paclitaxel (pPTX) and polymeric β-CD containing nitric oxide (pCD-pSNO) through host-guest interactions (Figure 6b) 92. In comparison to free reagents alone or in combination, the prepared pPTX/pCD-pSNO exhibited significantly enhanced cytotoxicity, immunogenic cell death, dendritic cell activation and, T cell expansion* in vitro*. When combined with T lymphocyte antigen-4, pPTX/pCDpSNO could significantly prolong the survival time of animals.

It has been previously reported that PDT mediated by pyropheophorbide a (PPa) could enhance immunogenicity of tumor cells, and promoted infiltration of cytotoxic T lymphocytes 97. Yu et al. developed a supramolecular prodrug nano-platform that could co-deliver PPa and JQ1 (bromodomain-extraterminal protein 4 inhibitor) prodrugs for combined photoimmunotherapy of pancreatic cancer (Figure 6c) 93. Nanoparticles were prepared by host-guest complexation between β-CD-grafted hyaluronic acid (HA-CD) and adamantane-based prodrugs (AD-SS-JQ1 and AD-SS-PPa). Importantly, β-CD could produce assembly effect with PPa, JQ1 and adamantane at the same time. It was observed that HA could actively target tumors by recognizing highly expressed CD44 on the surface of pancreatic tumors. PPa-mediated PDT enhanced the immunogenicity of tumor cells and promoted the intratumor infiltration of T lymphocytes. At the same time, JQ1 resisted PDT-mediated immune evasion by inhibiting the expression of c-Myc and PD-L1. c-Myc and PD-L1 are key regulators that are known to be involved in tumor glycolysis and immune evasion.

It is generally believed that chemotherapy drugs will suppress immune system. However, a large number of studies showed that immune activation induced by chemotherapy could be used as an effective supplement to immunotherapy 98. Effective chemotherapy combined with immunotherapy strategies usually involve induction of immunogenic cell death to promote antigen presentation, elimination of immunosuppressive factors, improvement of immune cell activity, and enhancement of immune-related factors 99. The ultimate goal of combination of immunotherapy with chemotherapy is to produce more effector T cells that could bind to antigen expressed by tumor cells to improve the overall anti-tumor efficacy and effectively prevent tumor recurrence and metastasis 100. The combined application of immunotherapy and other therapies has broadened application prospects. However, some problems still need to be solved for specific applications. In particular, it is important to gain deeper understanding regarding the mechanisms involved in the interaction of chemotherapy, immunotherapy, and phototherapy, which would further help to accurately select combination treatment options. Additionally, the combination therapy should take into account side effects, such as bone marrow suppression by chemotherapy drugs, cytotoxicity associated with photosensitizers, and adverse reactions related to immunotherapy. It is important to optimize dosage, timing, and route of the combination therapy to reduce adverse reactions.

## 7. Co-transport of protein and others

Protein therapy is one of the important treatment strategies for cancer 101. When compared with small molecule drugs, protein therapy exhibits higher specificity and better biocompatibility. Various therapeutic proteins, including cytokines, enzymes, growth factors, and monoclonal antibodies, have been widely explored for cancer treatment. However, the co-delivery systems based on cyclodextrin for protein and others have not been widely studied (Table 6).

**Figure 7:**
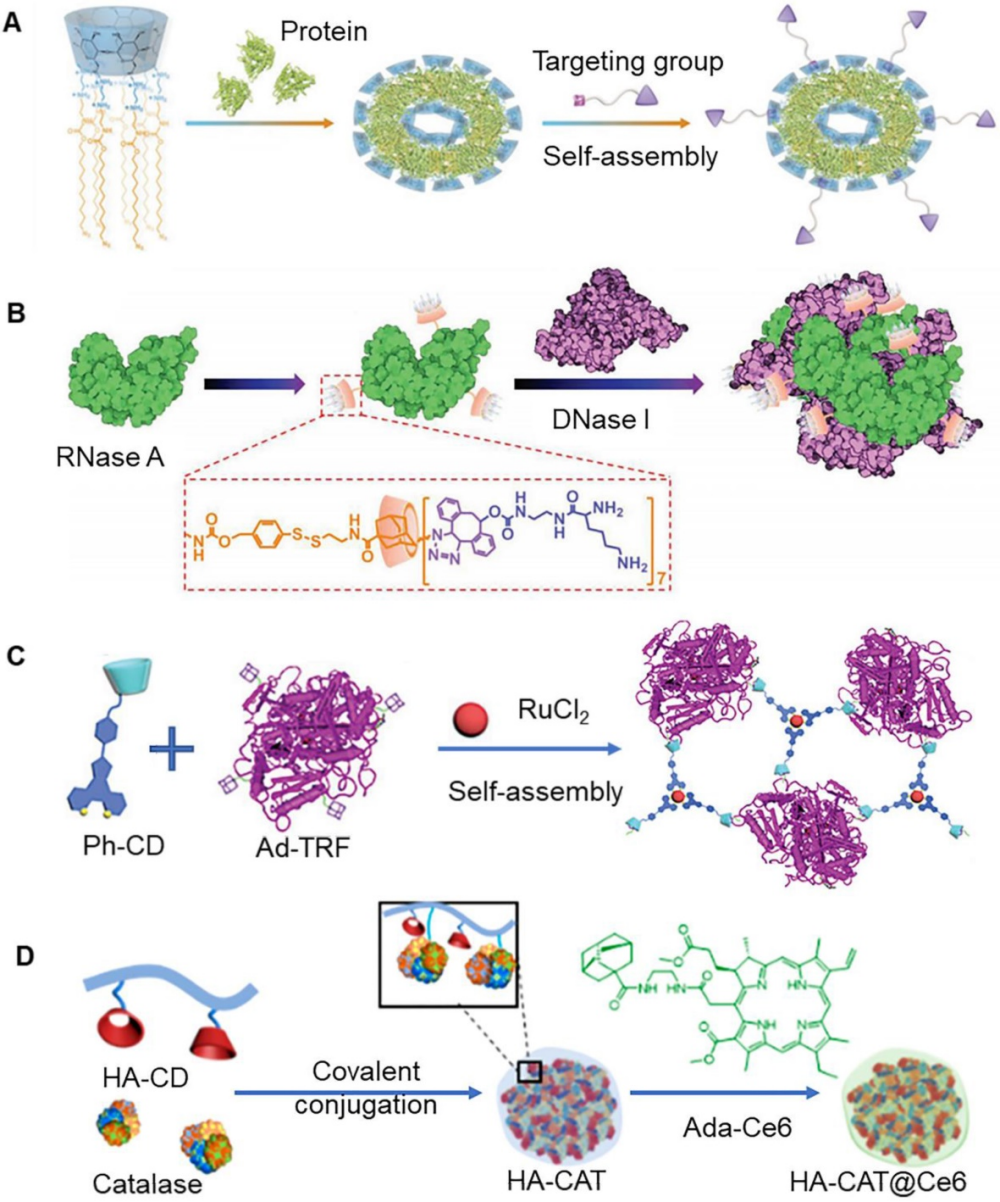
** (A)** Schematic of multiarmed amphiphilic cyclodextrin nanoparticles. Adapted with permission from 102, Copyright 2019 Wiley-VCH. **(B)** Assembly process of RNase A and DNase A. Adapted with permission from 103, Copyright 2021 Wiley-VCH. **(C)** Assembly process of three kinds of blocks: Ad-TRF, ruthenium and Ph-CD. Adapted with permission from 105, Copyright 2019 Royal Society of Chemistry. **(D)** Schematic of catalase and Ce6 coloaded nanoparticles. Adapted with permission from 106, Copyright 2019 American Chemical Society.

In the past few years, protein therapy attracted immense attention, primarily owing to its great potential in the treatment of various diseases 107. However, issues of poor stability and membrane impermeability associated with protein drugs limit their clinical application. To address these issues, it is important to develop a more efficient protein delivery system. In a previous study, Feng et al. reported the construction of multiarmed amphiphilic β-CD (CDEH) nanoparticles that could co-delivery proteins and genes to tumor cells (Figure 7a) 102. In addition to this, these nanoparticles could be further functionalized with targeting molecule on the base of non-covalent interaction between β-CD and guest molecules. It was observed that folate modified nanoparticles could also deliver (CRISPR)/Cas9 protein and *sgRNA* into HeLa cells, resulting in specific gene deletion and inhibition of tumor growth in HeLa tumor bearing mice. In addition to single protein therapy, multiple functional protein combination therapy has been shown to provide a new strategy for cancer treatment. As shown in Figure 7b, Ribonuclease A (RNase A) modified by adamantane could self-assemble with lysine-β-CD to form nanoparticles 103. These nanoparticles exhibited cationic characteristics, which enhanced stability and cellular uptake, and assisted in binding to deoxyribonuclease I (DNase A). Importantly, disulfide bond present in the nanoparticles could be broken under specific environmental conditions in the tumor, which resulted in the release of RNase A and DNase A enzymes into the tumor cells. These two enzymes could further degrade RNA and DNA, respectively. The protein co-transport strategy can also be applied to other functional proteins, which hold great significance in the treatment of tumors.

A previous study reported that 6-arm PEGylated crystalline β-CD could be assembled with IR825 dye to form nanoparticles, through host-guest interaction. It was characterized by an excellent ability to encapsulate proteins (INFα) 104. Importantly, IR825 in the nanoparticles could promote protein release following near-infrared illumination, which could be used to enhance cancer treatment. Transferrin protein can also be modified with adamantan (Ad-TRF). A previous study reported that Ad-TRF, ruthenium, and phenanthroline-modified β-CD (Ph-CD) could form multifunctional nanoparticles through self-assembly interaction (Figure 7c) 105. These nanoparticles exhibited specific ability to target tumor cells. Additionally, these nanoparticles demonstrated high photodynamic therapy ability under visible light irradiation. Furthermore, the nanoparticles showed selective killing of tumor cells and negligible toxicity towards normal cells. It has been previously shown that catalase could catalase could enhance the effect of photodynamic therapy on tumors 108. Adamantane-modified chlorin e6 (Ada-Ce6) could be assembled with β-CD-grafted hyaluronic acid (HA-CD) to form nanoparticles, which could co-deliver catalase to tumor cells (Figure 7d) 106. Catalase is known to catalyze endogenous hydrogen peroxide to generate oxygen. During photodynamic therapy, oxygen could be further converted into toxic singlet oxygen to kill tumor cells.

Transporting proteins into cells is a key technology involved in protein therapy. These delivery technologies have been widely used for cancer treatment, regenerative medicine, vaccination, and diagnosis. In the past few years, more and more protein therapies have been developed 109. When compared with small molecule drugs, protein therapy offers the advantages of strong specificity and complex functions, and thus can be considered as a safe alternative to gene therapy 110. However, there are certain challenges. At present, most of the studies provide short-term hematological and pathological results, which are not enough for clinical application of protein-based nano medical systems. In addition to this, other important factors, such as potential immunogenicity, might need to be considered. Some special populations might produce immune responses towards specific proteins in a short or long time, which presents an important challenge for the utilization of protein-based nanomedicine.

## 8. Transport of theranostic agents

Theranostic refers to a process, wherein therapy and diagnosis can be performed simultaneously. Integration of therapy and diagnosis usually requires an efficient co-transporter to simultaneously transport diagnostic and therapeutic agents to the tumor site for disease diagnosis, therapy, and timely evaluation of the effect of therapy. CDs have been widely used to construct carriers for co-transportation of therapeutic and diagnostic agents, primarily owing to ease of modification and host-guest interaction properties (Table 7).

**Figure 8:**
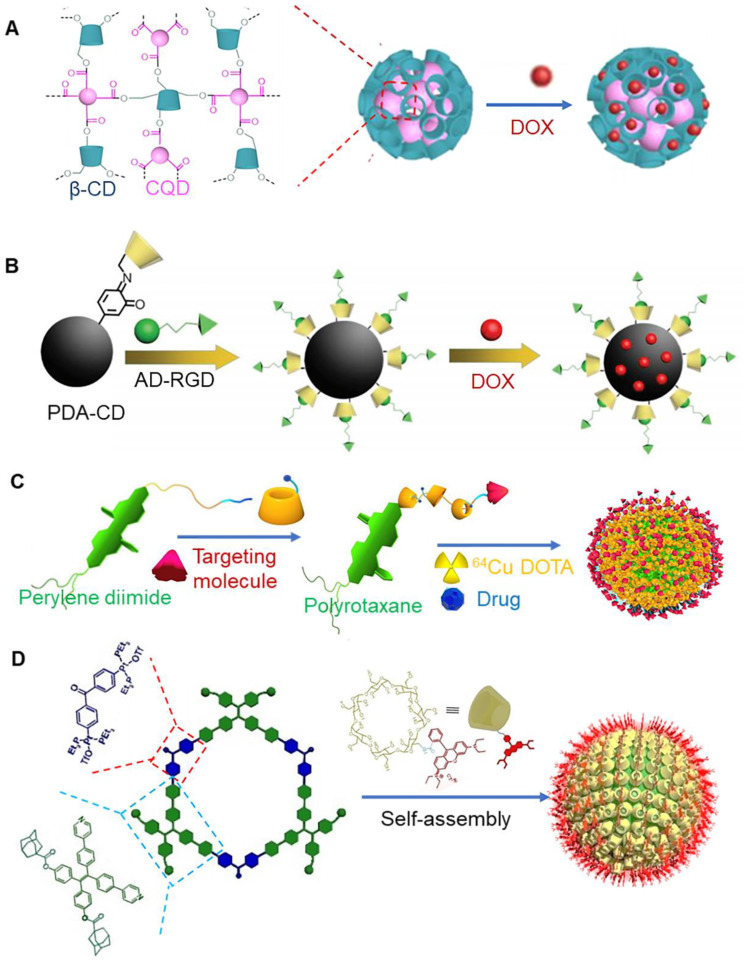
** (A)** Schematic of carbon quantum dots based on cyclodextrin nanoparticles. Adapted with permission from 112, Copyright 2018 American Chemical Society. **(B)** Assembly process of polydopamine nanoparticle based on cyclodextrin. Adapted with permission from 114, Copyright 2018 Wiley-VCH. **(C)** Assembly process of cyclodextrin polyrotaxane carrier. Adapted with permission from 115, Copyright 2018 Springer Nature. **(D)** Assembly process of fluorescent metallacycle-cored amphiphilic nanoparticles. Adapted with permission from 117, Copyright 2020 Wiley-VCH.

Quantum dots (QDs) are reliable diagnostic technologies that have been widely used for tumor imaging and drug delivery tracking. A previous study reported that α-CD-modified doxorubicin prodrugs (CD-DOX) and alkyl chain-modified ZnAgInSe/ZnS (ZAISe/ZnS) quantum dots could self-assemble to form nanoparticles, which was mediated via host-guest interaction between α-CD and alkyl chains. These nanoparticles further interacted via charge interactions, and anionic folic acid covered the surface of nanoparticles 111. Carbon quantum dots (CQDS) represent a new type of fluorescent carbon nano materials. These nano materials offer advantages of high stability, good biocompatibility, ease of synthesis and surface functionalization, and unique optical properties. CQDS have been widely used in biological imaging. Carboxyl-functionalized carbon quantum dots are known to exhibit high quantum yield and abundant surface carboxyl groups, which could be used as cross-linking agents for β-CD, endowing fluorescent functions to β-CD (Figure 8a) 112. This cross-linked polymer has been shown exhibit both fluorescence imaging properties and drug delivery functions. Importantly, it finds good application prospects in diagnosis and treatment of tumors.

Ruthenium (Ru) polypyridyl complexes have been widely used as biological imaging agents in the past few years. A previous study reported that adamantane functionalized fluorescent Ru complexes and dimer polymer (cyclodextrin thioketal cyclodextrin) could be self-assembled to form nanoparticles with therapeutic and diagnostic functions. Anticancer drugs camptothecin and vitamin K_3_ could be effectively loaded together in these nanoparticles. Interestingly, vitamin K_3_ present in the carrier quenched red fluorescence of Ru polypyridyl complexes, once released 113. Importantly, the release process of the drugs from the nanoparticles could be easily tracked in terms of fluorescence changes. Phosphorescent iridium(Ir) complexes are known to exhibit dual functions as anticancer agent and in imaging 118. Polydopamine nanoparticles offer good biocompatibility, near infrared absorption, and photothermal effect. Its surface can be modified by β-CD. A previous study reported that adamantane modified RGD tripeptides could be assembled with β-CD to make polydopamine nanoparticles that exhibited tumor targeting ability (Figure 8b) 114. When phosphorescent iridium was complexed with these nanoparticles, the nanoparticles could be applied for targeted combined cancer photothermal-chemotherapy and thermal/photoacoustic/two-photon phosphorescence lifetime imaging.

A previous study demonstrated that β-CD polyrotaxane carrier could transfer drugs, contrast agents (^64^Cu DOTA), and photothermal agents (perylene diimide) to tumor sites (Figure 8c) 115. Importantly, β-CD in polyrotaxane could modify contrast agent to monitor transmission and metabolism of nano drugs *in vivo*. The axis of polyrotaxane was amphiphilic in nature, and it could be used to load anticancer drugs. Perylene diimide, a capping agent, was endowed with the ability to absorb near-infrared light and convert it into heat, which would accelerate the release of drugs into cancer cells. In addition to this, photothermal therapy by itself could effectively kill tumor cells and form a synergistic effect with chemotherapy.

Aggregation induced emission (AIE) phenomenon has been widely used in the field of tumor imaging. A previous study reported the use of a conjugate of AIE active compound (phenylboronic acid) and hyperbranched polyglycerol functionalized β-CD as a drug carrier 116. This cyclodextrin-based AIE-active composites exhibited great potential to be used in bioimaging and drug delivery applications. Tetraphenylethylene (TPE) is widely used as AIE active compound 119. TPE could form fluorescent metallacycles after binding with Pt^II^ ligands 120. Zhang et al. constructed fluorescent metallacycles with pendent adamantane groups, and then self-assembled with rhodamine-modified β-CD to form nanoparticles (Figure 8d) 117. β-CD could enhance the water solubility and bioactivity of metallacycles, making the formed nanoparticles with amphiphilic properties. Due to the efficient fluorescence resonance energy transfer from the metallacycles to rhodamine, the release of the metallacycles from the nanoparticles could be monitored *in situ* by changes in fluorescence. The nanoparticle can not only be used as cell imaging contrast agents, but also has good anticancer activity, which is a potential diagnostic and therapeutic platform.

For nanocarriers, the accuracy of imaging results is critical for precise diagnosis and subsequent treatment of cancer. The currently available nanocarriers are not very accurate for tumor localization, especially for early-stage tumors and metastatic lesions. Thus, there is need to design nanocarriers that are sensitive to tumor cell metabolic markers and enzymes. These tumor-specific substrates might assist in the imaging of diagnostic agents by tuning non-covalent bonds.

## 9. Conclusion and perspectives

As discussed above, multifunctional co-delivery systems based on cyclodextrin have been widely developed and applied in the field of cancer therapy. Importantly, the host-guest chemistry can be utilized to conveniently construct multifunctional co-delivery systems with lot of ease and flexibility. Various small molecules can be self-assembled to produce multifunctional supramolecules through host-guest chemistry, usually from inside to outside, from bottom to top, and from small to large. Importantly, rational utilization of stimulus response characteristics of the host and guest chemistry would aid in controlling space and time of release of the loaded cargo. This in turn would greatly improve therapeutic effect and reduce side effects. Multifunctional co-delivery systems based on cyclodextrin assembly have been extensively developed in the past few years, and these systems have achieved a series of remarkable progress. However, some issues need to be further explored and resolved in the future.

Biological safety presents a particularly important concern in the designing of these nanocarriers. If these artificial compounds cannot be removed quickly and effectively from the body, cytotoxicity will be unavoidable. Thus, more attention is required during the development of new multifunctional co-transport carrier systems. Next-generation multifunctional carriers need to exhibit biocompatibility and degradability, while maintaining host-guest complexation ability and stimulus response ability.

Currently, the modification of cyclodextrin is mainly limited to mono-substitution on the main surface. There are few studies that reported regioselective modification of cyclodextrin. Therefore, it is necessary to establish more efficient synthetic strategies to synthesize various substituted cyclodextrins. In addition to this, different structures of the assembly system should be designed to enrich the applications of cyclodextrins in more co-delivery carriers. Reasonable designing and manufacturing of carrier is very important. Importantly, the carrier system that can be transformed into clinical application needs to be simple and reliable. Cyclodextrin based multifunctional nanocarriers often exhibit complex structures and manufacturing steps, which might lead to poor repeatability and reliability. Future studies should focus on utilization of advantages of self-assembly to prepare multifunctional nanocarriers by "one-pot method".

During delivery process and following cellular internalization, it is important to reasonably balance the assembly and disassembly abilities of co-transporter based on the host-guest system. In case the ability to assemble is too strong, it is not conducive for the release of cargo, present inside the carrier, in the cell. However, in case the ability to assemble is too weak, the cargo would be released in advance during the transportation process. A reasonable assembling ability is known to be beneficial for enhancement of the anti-cancer effect and reduction of adverse side effects. It has been previously reported that when association constants were lower than 10^5^ M^-1^, the nanocarriers usually disassembled due to environmental dilution, after entering the systemic circulation 26. Currently, the assembly of cyclodextrin with only a small amount of guest molecules (ferrocene and benzimidazole) has the ability to respond to tumor microenvironment 17. In addition to this, the sensitivity of this response ability is not very high. Besides this, it cannot be easily disturbed. The difference between tumor microenvironment and normal environment is usually small, so it is necessary to study highly sensitive host-guest molecules, particularly to identify environmental differences and controlled release behavior of the carrier in a better way.

In order to achieve efficient tumor treatment, it is important to successfully deliver therapeutic agents to specific subcellular locations (nucleus, mitochondria, lysosome, and others). Cyclodextrin based delivery systems mainly focus on cell membrane targeting. However, few studies are available on subcellular targeting ability. Currently, only limited knowledge is available regarding intracellular transport of subcellularly targeted nanocarriers and their time-dependent fate and release profile of therapeutic agents 121. In order to realize the direct entry of therapeutic agents into subcellular locations, the designs based on cyclodextrin carrier might be more complex.

In the view of complexity of tumor treatment, it is important that experts from different research fields, such as cancer biology, chemistry, nanoscience, materials science, and pharmacy, comprehensively and powerfully cooperate to design and construct a combined treatment system. Importantly, there is an urgent need to identify new biological materials to develop personalized treatment platforms for precise diagnosis and intelligent treatment. Altogether, the present review highlighted that cyclodextrin‐based supramolecular self-assembly chemistry provide new options for the construction of intelligent and effective nano-therapeutic systems. In the view of ongoing research, it is believed that there would be major breakthroughs in cyclodextrin-based supramolecular self-assembly therapy platform in the future, especially in the field of cancer therapy.

## Figures and Tables

**Table 1:** Basic information of co-delivery systems (drug and drug).

Host:Guest	Other Components	Therapeutic Agents	Reference
β-CD: Celecoxib	MSN	Celecoxib, DOX	31
β-CD: Adamantane	Fe_3_O_4,_ MSN	Pt, DOX	32
β-CD: Paclitaxel	Au, MSN	Paclitaxel, DOX	33
β-CD: MTX	Graphene, Fe_3_O_4_	MTX, DOX	34
β-CD: Curcumin	Fe_3_O_4_, hydrogels	Curcumin, DOX	35
β-CD: Aminosalicylic acid-butyric acid	PEG	Aminosalicylic acid, Butyric acid	36
β-CD: Curcumin-Pt	Hyaluronic acid	Curcumin, Pt	37
β-CD: Oxaliplatin, γ-CD: Dox	Epichlorohydrin	Pt, DOX	38

**Table 2:** Basic information of co-delivery systems (drug and regulatory gene).

Host:Guest	Other Components	Therapeutic Agents	Reference
β-CD: Monensin	PEI	Monensin, TRAIL DNA	48
β-CD: Adamantane	PEI	DOX, siRNA	49
β-CD: DOX	PDMAEMA	DOX, miR-122	50
β-CD: DOC	Poly(amido amine)s	DOC, TFPI-2	51
β-CD: DTX	Hydrocarbon chains, Polar (propylamino)	DTX, siRNA	52
β-CD: Adamantane, β-CD: DOX	PDMAEMA	DOX, Nur77ΔDBD	53
β-CD: SN-38	Polyamidoamine, MSN	SN-38, siRNA	54

**Table 3:** Basic information of co-delivery systems (drug and photosensitizer).

Host:Guest	Other Components	Therapeutic Agents	Reference
β-CD: CPT	β-NaYF_4_, MSN, Lactobionic acid	CPT, DHMA	60
β-CD: DOX	PEG	DOX, Zinc phthalocyanine	61
β-CD: Adamantane	Hyaluronic acid	CPT, Porphyrin	62
β-CD: DOX	PEG	DOX, Tungsten nitride	63
β-CD: Adamantane	Hyaluronic acid	Naphthalimide, CPT, IR825	64
β-CD: Adamantane	Pyridine amphiphilic	Pt, IR825	65

**Table 4:** Basic information of co-delivery systems (regulatory gene and photosensitizer).

Host:Guest	Other Components	Therapeutic Agents	Reference
β-CD: Fc	Polycation	p53 gene, TAPc	76
β-CD: TPP	Poly(L-lysine)	p-DNA, TPP	77
β-CD: Adamantane	Poly (glycidyl methacrylate)s	p53 gene, Killerred protein	78
β-CD: Adamantane	Au, MSN, Poly (glycidyl methacrylate)s	p-DNA, Au, DOX	79
β-CD: Adamantane	Au, Poly (glycidyl methacrylate)s	p-DNA, Au	80

**Table 5:** Basic information of co-transporter assemblies (immunopotentiator and drug (protein or photosensitizer)).

Host:Guest	Other Components	Therapeutic Agents	Reference
β-CD: Adamantane	PHEA, PAMAM	CpG, Pt	88
α-CD: DOX	Polyamidoamine	CpG, DOX, Indocyanine green	89
β-CD: R837	Mannose	Trp2, R837	90
β-CD: Quercetin	PEG	Rg3, Quercetin	91
β-CD: PTX	Poly(maleic anhydride)	PTX, Nitric oxide	92
β-CD: Adamantane	Hyaluronic acid	JQ1, Pyropheophorbide a	93

**Table 6:** Basic information of co-delivery systems (protein and protein (gene or photosensitizer)).

Host:Guest	Other Components	Therapeutic Agents	Reference
β-CD: Adamantane	Hydrocarbon chains	(CRISPR)/Cas9 Protein, sgRNA	102
β-CD: Adamantane	Lysine	Ribonuclease A, Deoxyribonuclease I	103
β-CD: IR825	PEG	INFα, IR825	104
β-CD: Adamantane	Polysaccharide	Protein, Ru	105
β-CD: Adamantane	Hyaluronic acid	Catalase, Chlorin e6	106

**Table 7:** Basic information of transporter assemblies (theranostic agents).

Host:Guest	Other Components	Therapeutic/imaging agents	Reference
α-CD: Alkyl chain	Alkyl chain	ZAISe/ZnS, DOX	111
β-CD: DOX	Carbon quantum dots	Carbon quantum dots, DOX	112
β-CD: Adamantane	polypyridyl	Ru, CPT, VK3	113
β-CD: Adamantane	Polydopamine	Ir, Polydopamine	114
β-CD: Poly(ε-caprolactone)	Poly(ε-caprolactone)	^64^Cu DOTA, Perylene diimide, PTX	115
β-CD: DOX	Hyperbranched polyglycerol	Phenylboronic acid, DOX	116
β-CD: Adamantane	Tetraphenylethylene	Tetraphenylethylene, DOX	117
